# AI-ECG for early diagnosis of atrial fibrillation: an under-utilized tool for preemptive stroke prevention

**DOI:** 10.1097/MS9.0000000000004403

**Published:** 2025-12-03

**Authors:** Azka Shahid, Hamza Sajid, Noor un nisa Irshad, Sakan Binte Imran

**Affiliations:** a Department of Medicine, Allama Iqbal Medical College, Lahore, Pakistan; b Department of Medicine, Jinnah Sindh Medical University, Karachi, Pakistan; c Department of Medicine, Sir Salimullah Medical College, Dhaka, Bangladesh

**Keywords:** AI-ECG, anticoagulants, artificial intelligence enabled ECG, atrial fibrillation, ischemic stroke, machine learning, stroke

*Dear Editor,*

Atrial Fibrillation (AF) is among the most common arrhythmias worldwide. It has been documented as causing as much as a five-fold increase in the incidence of stroke^[1]^. In individuals with AF without anticoagulant therapy, the incidence of stroke ranges from 7.7 to 30.8 per 1000 person-years^[2]^. Conventional therapy for AF involves treatment with anticoagulants, which significantly reduce stroke events. A 64% decrease in stroke incidence was observed with the use of oral anticoagulants (OACs) like warfarin, with a further 19% decrease upon using Novel Oral Anticoagulants (NOACs)^[3]^. Artificial intelligence-enabled ECG (AI-ECG) has emerged as a promising diagnostic technique for silent or paroxysmal atrial fibrillation, with potential implications for stroke prevention. Early diagnosis of silent AF enables the timely preventative use of anticoagulants to reduce stroke risk. A recent clinical trial implemented an AI-ECG-based alert system for the prescription of oral anticoagulants in patients with AF^[4]^. While this is a step in the right direction, most studies on AI-ECG emphasize performance metrics and rarely report clinical impact, leaving a significant gap between AI-ECG in research and its real-world implementation.

Recent advancements in AI-ECG models have demonstrated increasing accuracy in the diagnosis of AF^[5]^. Beyond trial evidence, retrospective analyses have shown that AI-ECG can identify AF signatures even during sinus rhythm, with an AUC of ~0.90 and sensitivity/specificity above 80%^[6]^. AI-ECG has excellent diagnostic performance, but improvement in patient outcomes is limited if it doesn’t translate to treatment changes. In a randomized pragmatic trial of AI-ECG alerts, physicians who received AI-ECG feedback for patients had significantly higher rates of non-vitamin K antagonist oral anticoagulant prescription (23.3% vs 12.0%; hazard ratio 1.85) than controls, but no significant reduction in ischemic stroke or cardiovascular death was observed over the trial period^[4]^. Similarly, very few studies have addressed the cost-effectiveness of AF screening with wearable devices^[7]^. Furthermore, challenges such as variable physician adoption, patient adherence to long-term anticoagulant therapy, and integration of AI outputs into clinical workflows remain largely underexplored^[8]^.

Management of AF does not end with detection alone; the end goal is the prevention of disabling and potentially fatal strokes through early diagnosis and appropriate anticoagulant use^[8]^. The real test of any AI model is whether it drives interventions that ultimately benefit patients^[9]^. Clinical benefits are limited if anticoagulants are not appropriately used after diagnosis. AI-ECG use also raises ethical concerns due to doubts that it may lead to overdiagnosis, causing patient anxiety, overburdened healthcare systems and overtreatment^[8]^.

To harness the full potential of AI-ECGs, future research must focus on clinical outcomes instead of diagnostic accuracy. Trials should assess whether AI-powered detection leads to increased oral anticoagulant initiation, long-term adherence, and reductions in stroke incidence. In a recent pragmatic trial, AI-ECG alerts were associated with higher rates of OAC prescription, but no reduction in stroke or cardiovascular death was observed, highlighting the need for long-term outcome data^[4]^. Integration into care pathways is essential, since algorithmic alerts alone are unlikely to change management without clinician decision support and patient engagement^[10]^. Cost-effectiveness analyses are also needed. Combining wearable technologies with AI-enabled ECG interpretation may lower costs by enabling large-scale, automated AF screening. Wearable AF screening devices could be cost effective, with one study putting it at $57 894 per quality-adjusted life-year^[7]^.

AI-ECG has the potential to advance AF detection, particularly silent and paroxysmal AF. The clinical value of AI-ECG cannot be proven solely based on diagnostic performance and accuracy. It must be supported by concrete evidence of improved patient outcomes, including timely anticoagulant initiation, long-term adherence to therapy, and reduction in stroke incidence. Current evidence demonstrates a gap between detection and intervention, along with limited data on cost-effectiveness and integration into care pathways. Outcome-oriented trials and real-world studies are therefore crucial to establish whether AI-ECG can reduce stroke burden at a global and population level. Until then, the role of AI-ECG should be viewed as promising yet provisional. This article aligns with the TITAN Guidelines on the need for transparency in AI use in healthcare^[11]^.

## Data Availability

Not applicable.
